# ADME-Space: a new tool for medicinal chemists to explore ADME properties

**DOI:** 10.1038/s41598-017-06692-0

**Published:** 2017-07-25

**Authors:** Giovanni Bocci, Emanuele Carosati, Philippe Vayer, Alban Arrault, Sylvain Lozano, Gabriele Cruciani

**Affiliations:** 1 Laboratory of Chemometrics, Department of Chemistry, Biology and Biotechnology, University of Perugia, Via Elce di Sotto 8, 06123 Perugia, Italy; 2 Technologie Servier, 25-27 rue Eugène Vignat, BP 11749, 45007 Orléans, cedex 1 France

## Abstract

We introduce a new chemical space for drugs and drug-like molecules, exclusively based on their in silico ADME behaviour. This ADME-Space is based on self-organizing map (SOM) applied to 26,000 molecules. Twenty accurate QSPR models, describing important ADME properties, were developed and, successively, used as new molecular descriptors not related to molecular structure. Applications include permeability, active transport, metabolism and bioavailability studies, but the method can be even used to discuss drug-drug interactions (DDIs) or it can be extended to additional ADME properties. Thus, the ADME-Space opens a new framework for the multi-parametric data analysis in drug discovery where all ADME behaviours of molecules are condensed in one map: it allows medicinal chemists to simultaneously monitor several ADME properties, to rapidly select optimal ADME profiles, retrieve warning on potential ADME problems and DDIs or select proper *in vitro* experiments.

## Introduction

The complex path of any new molecular entity (NME) to reach its target often involves the passage through several barriers as well as the survival into complicated biological systems. An ensemble of processes determine the bioavailability of a NME, and several factors may critically affect its pharmacokinetic (PK) properties. In the development of pharmaceutical drugs, this caused a high attrition rate: in the past, around 40% of all drug failures were due to adsorption, distribution, metabolism and excretion (ADME) problems^1^. Including preclinical ADME studies led to a reduction of failures caused by PK, but drug toxicity remains a problem^2, 3^. Both non-optimal ADME and toxicity (ADMET) can end up with late-stage failures, responsible for a big waste of time and money, and unfortunate cases like rofecoxib (Vioxx) and troglitazone (Rezulin) prompted the paradigm “fail early, fail cheap”^4^.

Parallel evaluation of efficacy and biopharmaceutical properties of drug candidates has been standardized, and exhaustive studies of ADME processes are nowadays routinely carried out at an early stage of drug discovery to reduce the attrition rate^5–7^. In order to help minimizing failures, computational strategies are still sought by biopharmaceutical researchers to predict the fate of drugs in the organism, and to identify early the risk of toxicity.

For this purpose, ADME-related in silico models are commonly used to provide a fast and preliminary screening of ADME properties before compounds are further investigated *in vitro*. Both private industry and academic researchers have extensively studied ADME-related properties, including the inhibition of the transporter P-glycoprotein (ABCB1 or Pgp) or enzymes of the cytochrome P450 (CYP) family, but also membrane permeability, volume of distribution or renal clearance^8–17^.

In our opinion, despite the utility of in silico models to predict ADME properties singularly, the lead optimization process would benefit of a simultaneous in silico study of several ADME properties, to go beyond the sum of the single models. We mean a unique model able to describe a drug pharmacokinetic profile in its whole, before *in vitro* experiments are carried out; it may be a space where molecules lie, and usable to investigate how structural changes might affect the ADME profile of a set of candidates. A model that can be used in multi-parametric optimization processes where ADME is often optimized in parallel to pharmacology.

Several ways to define and navigate (chemical) spaces appeared in the literature in the years, the most important being those based on structural descriptors^18–21^. The complexity of a chemical space needs algorithms for dimensionality reduction, for a simplified representation of the matrix of descriptors. For this purpose, principal component analysis (PCA) or artificial neural networks (ANN) algorithms are used the most often.

Like several chemoinformatic applications, the core concept of chemical space-based approaches is that similar molecular structures (i.e. points in the space with short distance between each other) often correspond to similar biological profile^21^. Therefore, new biologically active molecules are expected to lie in close proximity of known-actives. Translating to ADME, for any specific property, regions of the space exist where molecules have optimal values. However, using for years such chemical space approach we have observed that, when dealing with several ADME properties, the molecular description often remains too stuck to structural features, without catching the changes in the ADME behaviour. In other words, our major difficulties when using a chemical space for ADME where first, to have a common chemical space explaining all ADME properties, and second, to deal with activity-cliffs (situations with large changes in potency that correspond to small changes in the molecular structures)^22, 23^. An alternative chemical space, based on BDDCS classes, was proposed using VolSurf based models and GTM map, but this was limited to ADME properties linked to the BDDCS classes^24^.

Here, we attempt to change perspective, by modulating how molecules are described. Our proposal consists in describing molecules by their predicted ADME properties (derived by in silico QSPR models) rather than by structural features (molecular weight, size, flexibility, etc.) or physicochemical properties (logP, logD, pK_a_, etc.). Hence, predictions on twenty accurate QSPR models, derived for important ADME properties, define the new space, here called “ADME-Space”. We used the Self-Organising Maps (SOM) algorithm^25^ to represent the space as a 2D map derived from thousands of molecules. We preferred the non-linear method SOM to a linear one because it compresses better the descriptors information, particularly in our case where descriptors (QSPR predictions) are categorical values. Our aim is a holistic monitoring of the ADME profile, and making the ADME-Space tool able to help medicinal chemists in the simultaneous optimization of different ADME properties, leading to hypotheses for more targeted *in vitro* experiments.

In this article, we introduce these new concepts to help navigating the ADME space. We will go through the ADME-Space development, from the QSPR models to the final map and its application.

## Results

### Overview

In our procedure, any given molecule undergoes projection on twenty QSPR models for different biological properties: the results of these projections compose the ADME profile, and provide its molecular description. From this, we obtain a position onto the SOM map, so that the molecule can be assigned to a node, and all the properties previously assigned to that node are valid for the projected molecule, too.

### In silico models

Models from public data included the inhibition of membrane proteins responsible for drug active transport (efflux: Pgp, BCRP; and influx: OCT2, OATP1B1), as well as the recognition by Pgp and BCRP. Two additional properties were modelled starting from public data, the type of clearance (either renal or metabolic)^10^ and the maximum recommended daily dose (MRDD)^26^. Models for implication or inhibition of CYPs were available at Servier (based on in-house data for specific CYP isoforms: implication of 1A2, 2D6, 3A4^11^ and inhibition of 1A2, 2C9, 2D6, 3A4), *in vitro* metabolic stability in rat, mouse and human, intestinal absorption predictions from Caco2 experiments and brain permeability in rodents. In all these cases, the datasets counted hundreds of molecules, whereas larger datasets (thousands of molecules) developed with in-house Servier data, complete the list of in silico models.

### Models development and validation

We used curated datasets from the scientific literature for all the properties (Pgp inhibition^13^, Pgp recognition^27^, OCT2 inhibition^28^, OATP1B1 inhibition^29^, Clearance^10^ and MRDD^26^) except for inhibition of and recognition by BCRP, for which we curated the collection by using data from several articles. Foundation of QSPR classification studies were the categorical classification of compounds, based on experimental measures as suggested by the authors of the curated collections (mostly IC_50_ or percentage of inhibition). From the original collections, we also took the training/test sampling, in order to compare our results with those of the authors of the original models (see Methods section and Table 1).

**Table 1:** In silico models information.

Model	Dataset (all)	Class “+1”	Class “−1”	Dataset (training/test, %training)	Algorithm	Source
PGP INHIB	1272	664 Inhibitors^a^	608 Non-inhibitors^a^	772/503, 61%	Consensus (RF,SVM,AB,ETC,LDA)	Public
PGP RECOG	925	444 Substrates^a^	481 Non-substrates^a^	805/120, 87%	Consensus (RF,SVM,AB,ETC,LDA,DT)	Public
BCRP INHIB	935	418 Inhibitors^b^	517 Non-inhibitors^b^	668/267, 71%	Consensus (RF,SVM,AB,ETC,GB)	Public
BCRP RECOG	385	193 Substrates^c^	192 Non-substrates^c^	288/97, 75%	Consensus (RF,SVM,ETC)	Public
OCT2 INHIB	392	196 Inhibitors^a^	287 Non-inhibitors^a^	312/80, 80%	Consensus (RF,SVM,AB,ETC,GB)	Public
OATP INHIB	911	194 Inhibitors^a^	717 Non-inhibitors^a^	685/226, 75%	PLS	Public
CLEAR	469	286 Hepatic clearance^a^	183 Renal clearance^a^	Internal 5-fold cross validation	Consensus (RF,SVM,AB,ETC,LDA,DT)	Public
MRDD	1191	591 Low MRDD^a^	600 High MRDD^a^	1046/145, 88%	Consensus (RF,SVM,ETC,LDA)	Public
CACO2 PERM	10,109	8374 Permeable^d^	1735 Non-permeable^d^	6741/3368, 67%	RF	Private
BRAIN PERM	307	208 Permeable^e^	99 Non-permeable^e^	78/282, 25%	PLS	Private
CYP1A2 PERC	465	82 High implication^f^	383 Low implication^f^	310/155, 67%	SVM	Private
CYP2D6 PERC	448	89 High implication^f^	359 Low implication^f^	299/149, 67%	SVM	Private
CYP3A4 PERC	1442	1366 High implication^f^	76 Low implication^f^	961/481, 67%	RF	Private
CYP1A2 INHIB	922	144 Inhibitors^g^	778 Non-inhibitors^g^	615/307, 67%	RF	Private
CYP2C9 INHIB	715	233 Inhibitors^h^	482 Non-inhibitors^h^	476/239, 67%	SVM	Private
CYP2D6 INHIB	479	161 Inhibitors^i^	318 Non-inhibitors^i^	319/160, 67%	SVM	Private
CYP3A4 INHIB	913	375 Inhibitors^i^	538 Non-inhibitors^i^	609/304, 67%	SVM	Private
METASTAB human	10,056	3774 Stable^j^	6282 Unstable^j^	6705/3351, 67%	SVM	Private
METASTAB rat	10,056	2892 Stable^j^	7164 Unstable^j^	6705/3351, 67%	SVM	Private
METASTAB mouse	7026	2671 Stable^j^	4355 Unstable^j^	4684/2342, 67%	SVM	Private

^a^Compound categories are the same originally assigned by the authors of the works where data come from. ^b^Compound categories thresholds for BCRP INHIB model are IC_50_ ≤ 15 µM or inhibition ≥50% for inhibitors and IC_50_ > 50 µM or inhibition <30% for non-inhibitors. ^c^Compound categories for BCRP RECOG model were assigned based on experimental transport proved for substrates and experimental lack of transport proved for non-substrates. ^d^Fraction absorbed >90% for permeable compounds and fraction absorbed <40% for non-permeable compounds. ^e^In silico prediction of the Blood Brain Barrier (BBB) permeability *in vivo* in rodent is based on experimental measurement of brain to plasma ratio of concentrations (Kp) *in vivo* at 2 time points after compound administration in rodents (Kp > 0,8 for permeable compounds and Kp < 0,2 for non-permeable compounds). ^f^CYP implication >70% for high implication compounds and CYP implication <10% for low implication compounds. ^g^IC_50_ < 2 µM for inhibitors and IC_50_ > 15 µM for non-inhibitors ^h^IC_50_ < 2 µM for inhibitors and IC_50_ > 8 µM for non-inhibitors ^i^IC_50_ < 1 µM for inhibitors and IC_50_ > 10 µM for non-inhibitors. ^j^Stable when metabolic stability >60% and unstable when metabolic stability <30%.

For public datasets, chemical structures were retrieved as SMILES from the original articles or, when not available, from PubChem^30^, whereas for private datasets structures are from the internal Servier database. SMILES were converted to 3D structures (sdf format) with the software Marvin v6.2.1^31^. Successively, the program MoKa^32, 33^ was used to generate the most abundant tautomer and protomer at pH 7.4 for each structure.

With the software VolSurf+ ^31^ we imported the molecular structures and created the X-matrix of molecular descriptors (detailed elsewhere)^34–37^, which underwent the supervised classification procedures described below.

For all the models, the response was of the type −1/0/+1 and several classifiers were applied. For public datasets, we tested various regression and classification methods, as implemented in scikit-learn (an open-source python library for data mining and analysis)^38^. Different classifiers were combined in a way that only the consensus of the different methods gives the final classification. Data coming from literature collections is always affected by problems of reproducibility: comparing data is not so trivial due to different experimental protocols used. In this complex arena, the consensus between models is expected to be more robust and more accurate in prediction^39, 40^. In particular, our approach of the consensus of models provides the exclusion (when projecting an external molecule) of doubtful cases, for which the prediction of different models disagree. In general, when combining different methods, the full agreement or the agreement of 80% of the models (for example, 4 out of 5) guaranteed a final class assignment as either “−1” or “+1”, whereas incertitude (as mentioned, doubtful cases with the agreement of only 3 out of 5 models) led to uncertain predictions (assignment as “0”).

For Pgp and BCRP inhibition and recognition, OCT2 inhibition as well as Clearance and MRDD we used a combination of some of the following methods: random forest (RF), support vector machines (SVM), ada boost (AB), linear discriminant analysis (LDA), gradient boosting (GB), decision tree (DT) and extra trees classifier (ETC). Instead, in the cases of OATP1B1 inhibition (public data) and brain permeability (private data) models, we used PLS, and the comparison of the predicted Y with given thresholds was used for the final class assignment.

All the models developed at Servier were based on internal data from routine experiments that underwent the same experimental protocol (see Methods section). We used Knime^41^ on the descriptors X-matrix obtained with VolSurf+, and the most promising methods were selected: RF for Caco2, implication of CYP3A4 and inhibition of CYP1A2, and SVM for all the others. For each model, an applicability domain was defined, and those compounds predicted out of the domain were assigned “0” as predicted class. Model performance was evaluated using a naïve external dataset and computing the accuracy for balances or Matthew’s coefficient for unbalanced dataset.

**Figure 1:**
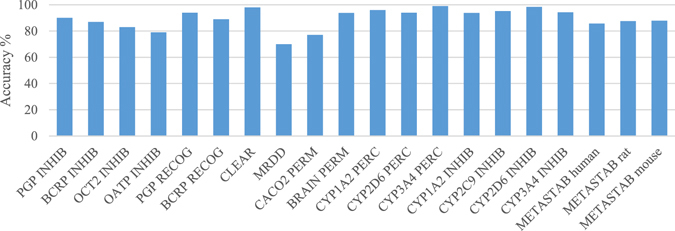
Accuracy values achieved by each model with external validation sets. For CACO2 PERM model, the reported value corresponds to the Matthew’s correlation coefficient.

The most relevant details of the models (which methods were used for the consensus) are given in Table 1, whereas models performance in external validation are shown in Fig. 1 and more technical details on the parameters used for each model can be found in Supplementary Information Tables S1–S7. In general, models are comparable with those developed in the original papers: if compared with the published performance, our results are at least as good in accuracy as the original ones. In several cases, the X-description was the same (VolSurf+) and we only changed the statistical treatment of the matrix, by combining classifiers and using the consensus of predictions. Thus, the expected good accuracy achieved by our models is not within the highlights of the present paper.

### ADME-Space development

Here, we introduce a new kind of descriptor: the predicted ADME properties; projections on single QSPR models are categorical values, with three values available for each property, high (“1”), low (“−1”) or uncertain (“0”). As an example, considering the inhibition model for Pgp, a molecule predicted as inhibitor assumes the value of “1”, a molecule predicted as non-inhibitor assumes the value of “−1”, whereas the value of “0” stands for those molecules the model was not able to classify, thereby labelled as uncertain. Table 2 reports the complete list of such classes.

**Table 2:** Prediction assigned arbitrary to each model categorical output.

Model	Predicted property value: “+1”	Predicted property value: “−1”
BCRP INHIB	Inhibitor	Non-inhibitor
BCRP RECOG	Substrate	Non-substrate
BRAIN PERM	Permeable	Non-permeable
CACO2 PERM	Permeable	Non-permeable
CLEAR	Metabolic	Renal
CYP1A2 PERC	High implication	Low implication
CYP1A2 INHIB	Inhibitor	Non-inhibitor
CYP2C9 INHIB	Inhibitor	Non-inhibitor
CYP2D6 PERC	High implication	Low implication
CYP2D6 INHIB	Inhibitor	Non-inhibitor
CYP3A4 PERC	High implication	Low implication
CYP3A4 INHIB	Inhibitor	Non-inhibitor
METASTAB human	Stable	Unstable
METASTAB mouse	Stable	Unstable
METASTAB rat	Stable	Unstable
MRDD	Low	High
OATP INHIB	Inhibitor	Non-inhibitor
OCT2 INHIB	Inhibitor	Non-inhibitor
PGP INHIB	Inhibitor	Non-inhibitor
PGP RECOG	Substrate	Non-substrate

If the output is “0”, the model was not able to classify the molecule, and the predicted class is “uncertain”.

To create the ADME-Space we used the self-organizing map (SOM) approach^25^. SOM is a type of artificial neural network that condenses the information contained in an *n*-dimensional matrix into a two-dimensional map where objects are clustered differently, based on their X-description. The SOM algorithm has been recently applied (by some of us) to define an applicability domain for UPLC-MS retention time prediction^42^, but also to several (and different) research fields, including structural sub-cluster analysis^28^, ligand-based virtual screening^43^ and docking-binding cavity analysis^44^.

Approximately 26,000 Servier molecules were extracted from an internal database, after filtering out compounds by molecular weight (retaining only if 100 < MW < 1000) and chemical composition (excluding those with elements other than C, H, N, O, P, S and halogens). A 50 × 50 map (2500 nodes) was built using the software “MaTCh” (Map The Chemicals), which is a Servier in-house implementation of the SOM algorithm. Though the low number of descriptors used, the high dimension (2500 possible positions) of the map is justified by the high number of molecules used for training. However, only 60% of the nodes of the ADME-Space were active (1498 nodes are labelled ON, because populated, i.e. contained at least one molecule) so carrying some information within. Vice versa, empty nodes (labelled OFF) do not contain information. Empty nodes are those having a vector too dissimilar from any molecule descriptors vector. Hence, they define portions of the space where molecules similar to those used to build it will never be found. Consequently, the ADME-Space applicability domain comprises the ensemble of nodes that are populated (ON nodes).

**Figure 2:**
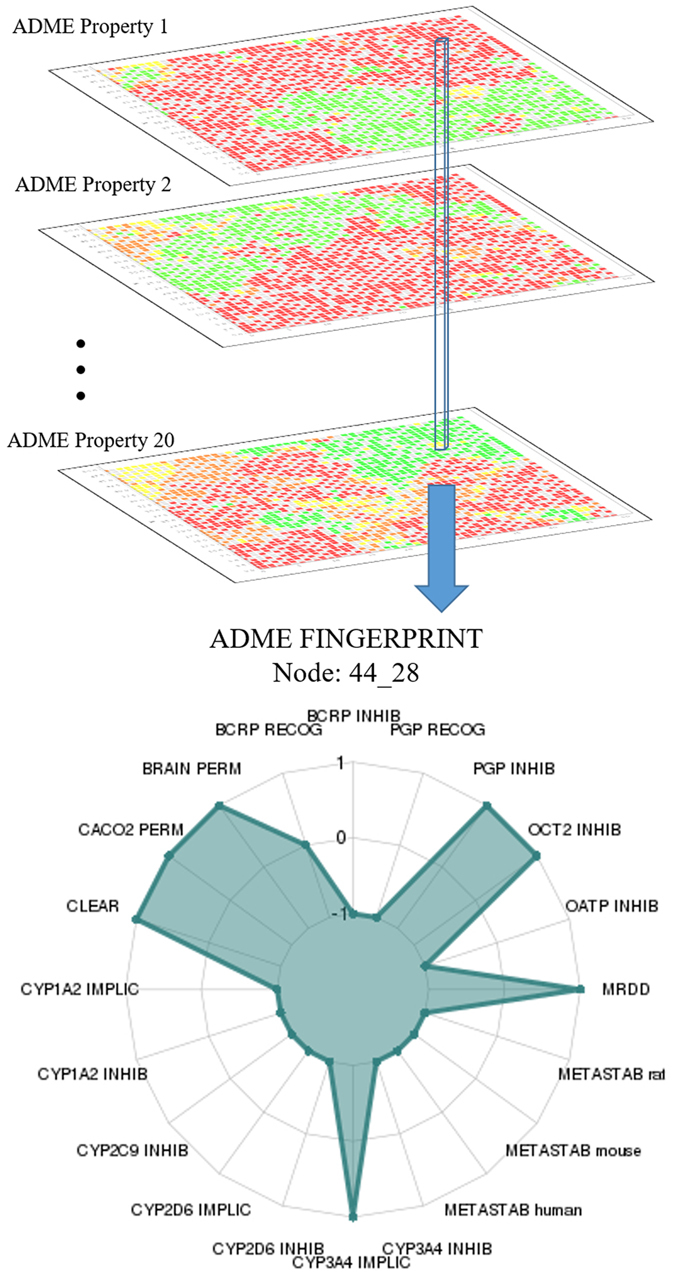
ADME fingerprint for the node 44_28. The multilayer representation of the ADME-Space (top) allows the comparison of the maps for more properties. The ADME fingerprint of a single node is the vector that goes through the maps for all the properties. It can be represented with a unique diagram (bottom), because for the given node the three values allowed correspond to the external circle (+1), the internal circle (0), and the central point (−1).

Given that we used 20 ADME properties to build the space, if considering two potential activity levels for any property (hence omitting the grey zone defined by the level ‘0’), an exhaustive set of all the possible combinations would count 2^20^, that is more than one million of different ADME fingerprints. With this perspective, the SOM map of ADME-Space is a powerful simplification, because 2500 nodes account for all the possibilities. Of course, there will exist molecules with ADME profile far from all the nodes: they will anyway be associated to a node, but the distance molecule-node would be big enough to force the method not to provide predictions for that molecule. These molecules can be seen as outliers compared to the set used to build the map. As well, nodes exist that are impossible to fill, because their vector is unrealistic (OFF nodes). They are “transition” nodes between realistic areas. A view of ADME-Space is as an ensemble of layers (see Fig. 2), with each layer being the distribution on the map for a specific ADME property. In all the layers, the nodes are colour-coded according to predicted property, and areas with defined borders are evident, where green nodes contain molecules with positive values, red nodes molecules with negative values, and yellow nodes represent areas of uncertainty (for the complete set of properties distribution plots, see Supplementary Information Figure S1). Given the perfect overlap of the layers, the vector that perpendicularly goes through all the layers defines the profile of each node. Figure 2 reports a graphical representation of the ADME fingerprint for the node “44_28”. For a given node, it is possible to know the number of molecules within the node (189 in this case) and to extract their profile. In this example, molecules are predicted with low metabolic stability in all the three species considered (human, rat and mouse), mainly due to CYP3A4 rather than other CYP isoforms, and the clearance is metabolic rather than renal. Other information concerns the high permeability in both Caco2 and brain, and low MRDD. Finally, there are two warnings for possible adverse drug reactions (ADRs) due to Pgp and OCT2 inhibition. Therefore, compounds predicted in this node should be further studied *in vitro* with Pgp and OCT2 inhibition assays, in order to verify the potential ADRs.

### ADME-Space validation

**Figure 3:**
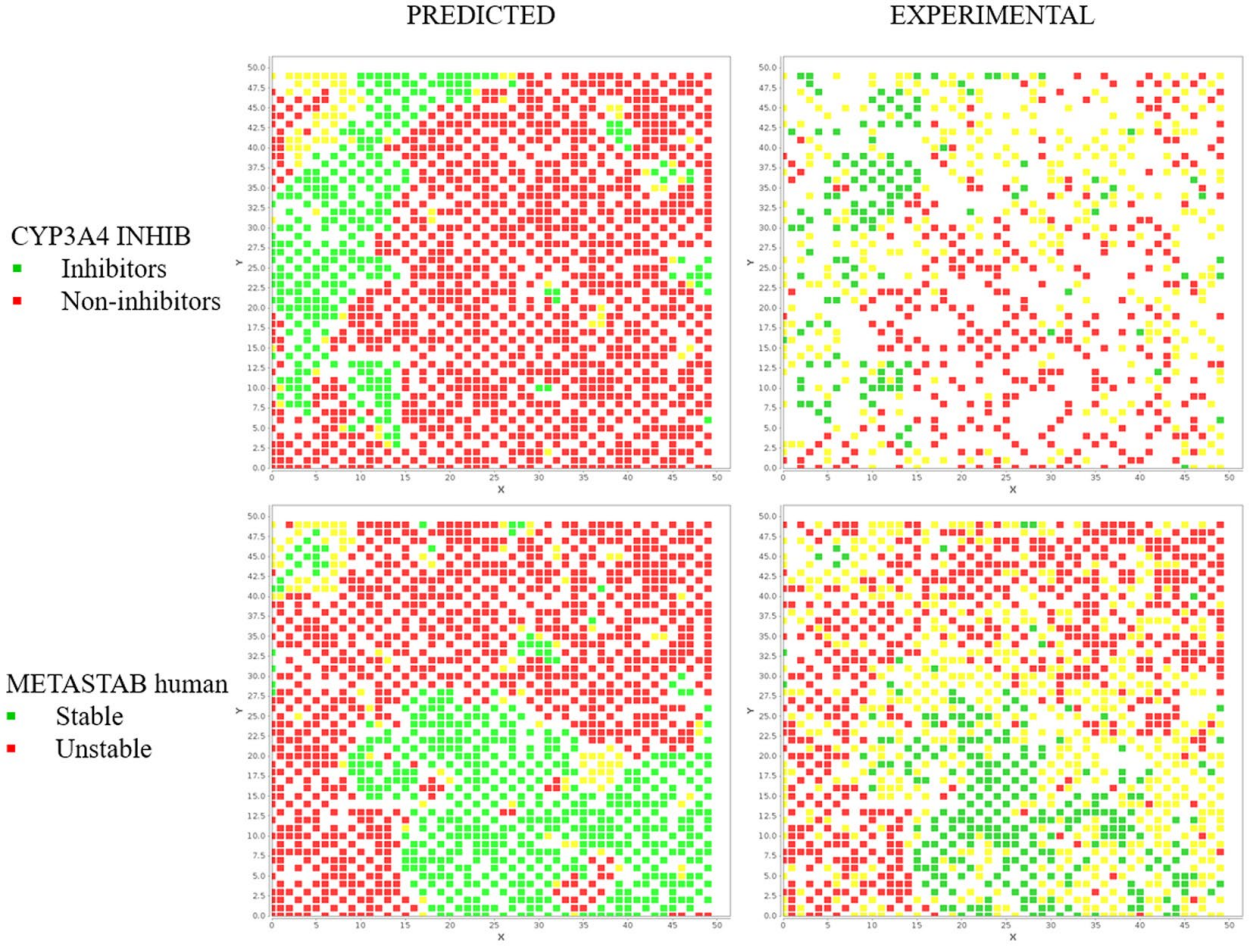
Comparison between predicted and experimental distributions for CYP3A4 INHIB and METASTAB human. Uncertain nodes are coloured in yellow.

The comparison between the distributions of predicted versus effective properties for the in silico models used can provide a fist idea of the space reliability. Real structures for which experimental data were known were projected in the map. Then they were coloured either by the in silico value predicted by the models or by the corresponding experimental value. As illustrated in Fig. 3 with metabolic stability in human and 3A4 inhibition, the shapes for predicted and experimental data are quite similar. This is due to the quality of models and it confirms the accuracy of descriptors as well. Furthermore, it suggests how experimental properties are wells distributed in the same map. Similar shapes of distributions were observed also for other properties.

### ADME-Space applications

Given the multilayer structure of the SOM, two or more ADME properties are easily comparable: this helps the simultaneous monitoring, necessary to guide the ADME optimization projects, in which we may focus on areas of the map characterized by no inhibition of cytochromes and transporters, along with low metabolism or high permeability, to mention a few.

Below we provide some examples to highlight how the ADME-Space can be used in drug development to decide which *in vitro* key ADME experiments may be carried out next. Routine ADME experiments often include metabolic stability, clearance and permeability, and give an overall idea of the most relevant processes. Besides these experiments, other important information may be necessary (implication of different cytochromes, mechanisms of absorption, influence of transport proteins), but the execution of the complete panel would increase the costs too much. Thus, warnings from the ADME-Space can guide the choice of additional experiments to perform, to minimize the research costs while optimizing the ADME profile of NMEs.

Finally, we used the ADME-Space to project external compounds, a small series of analogues designed to inhibit the bacterial efflux pump NorA, and a set of compounds having measured experimental solubility (soluble and not-soluble molecules). The results obtained are discussed below.

### Application 1: focus on permeability and active transport

**Figure 4:**
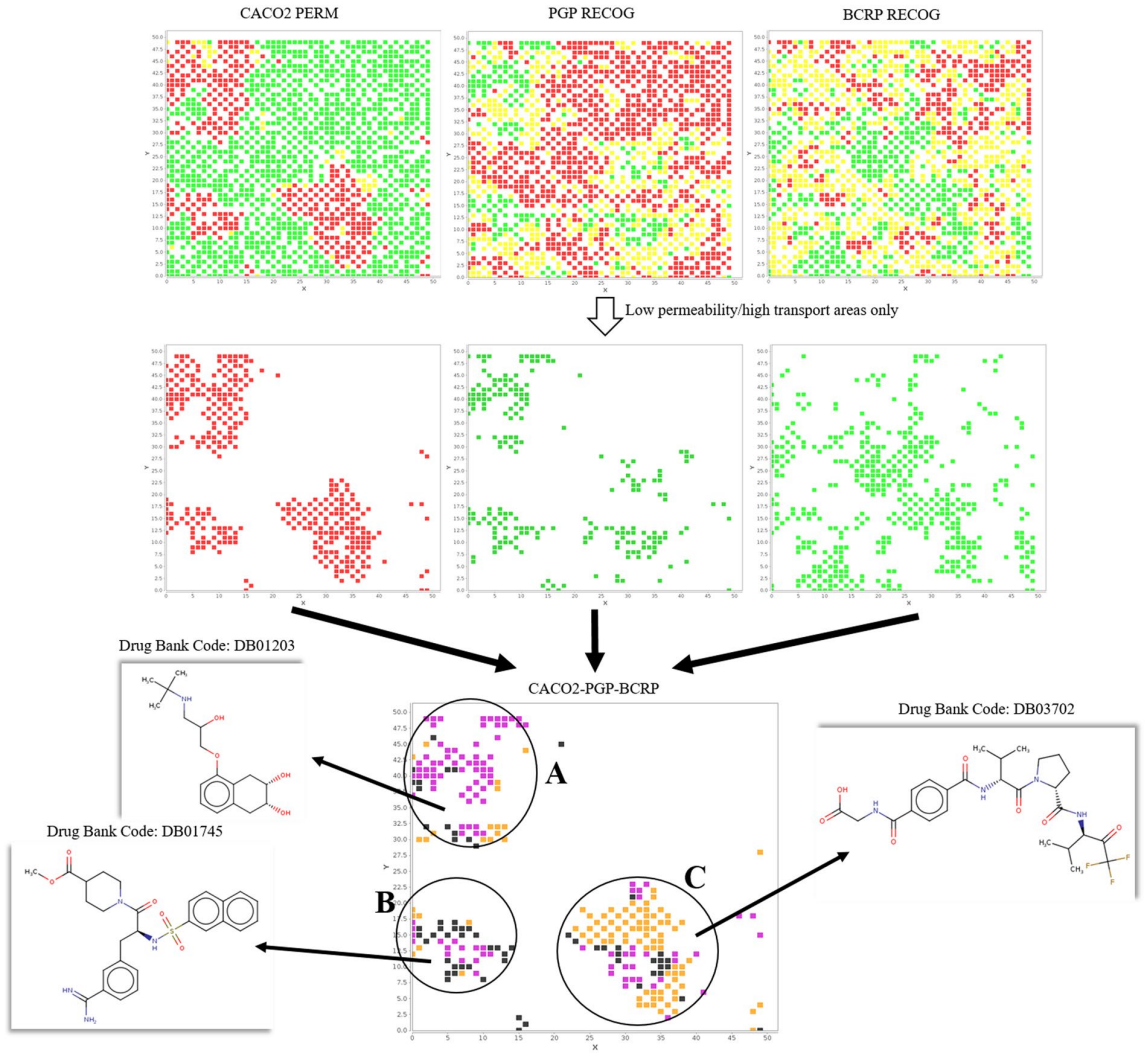
The first row of diagrams represent the SOM distribution for Caco2 permeability (green: permeable; red non-permeable), PGP recognition (green: substrate; red: non-substrate), and BCRP recognition (green: substrate; red non-substrate), whereas yellow nodes indicate where the property could not be defined (uncertain). Diagrams in the second row were obtained by filtering those of the first row: we kept only the nodes with low permeability (red) or high active transport (green, PGP and BCRP substrates). The content of the maps is merged, and we report in the final diagram three regions, marked with the codes (**A**–**C**). These contain nodes that match Caco2 low permeability and PGP/BCRP high transport, and different colours allow seeing which proteins are involved: purple nodes for Pgp transport only, orange nodes for BCRP transport only, black nodes for both proteins.

Efflux proteins can modulate the drug intestinal absorption, estimated *in vitro* by using Caco-2 cells. Figure 4 compares the maps of Caco-2 permeability and transport mediated by Pgp and BCRP. Noteworthy, we observed a similar profile of the maps for low permeability (red) and high transport (green) by Pgp. The profile of BCRP is different, but some intersecting regions exist. Thus, we identified three main areas (circled in black and marked as A, B and C), that correspond to low permeability regions, coloured by the efflux protein involved. As example, three public molecules, taken from DrugBank^45^, projected on the space and located in these three regions are reported. For the molecule DB01203, DrugBank reports low absorption and Pgp transport in accordance to our findings. The other two are experimental drugs for which no further ADME information is available. According to the maps, Pgp can be hypothesized as the major cause for the low permeability of compounds of the A region (purple cells), and the same can be hypothesized for BCRP (orange cells) for compounds of the C region. On the other hand, the low permeability of compounds from region B may be seen as a mixed effect of both transporters (black cells). In general, this result is in agreement with the experimental finding whereby low permeability molecules in Caco-2 experiments could be effectively transported by transport proteins^46^. Common output of Caco-2 experiments is whether active transport is involved, but not which protein is responsible. Thus, the maps can guide to run experiments toward Pgp, BCRP, both proteins or even none of them. Further evidence from the maps is the only partial overlapping of Pgp and BCRP maps that reflects a substrates diversity^47^. We also observed, in minor extent, mismatching regions (not shown), where the low permeability of molecules is not a consequence of efflux proteins, but could be due to other factors (such as, simply, high polarity).

### Application 2: focus on metabolism

**Figure 5:**
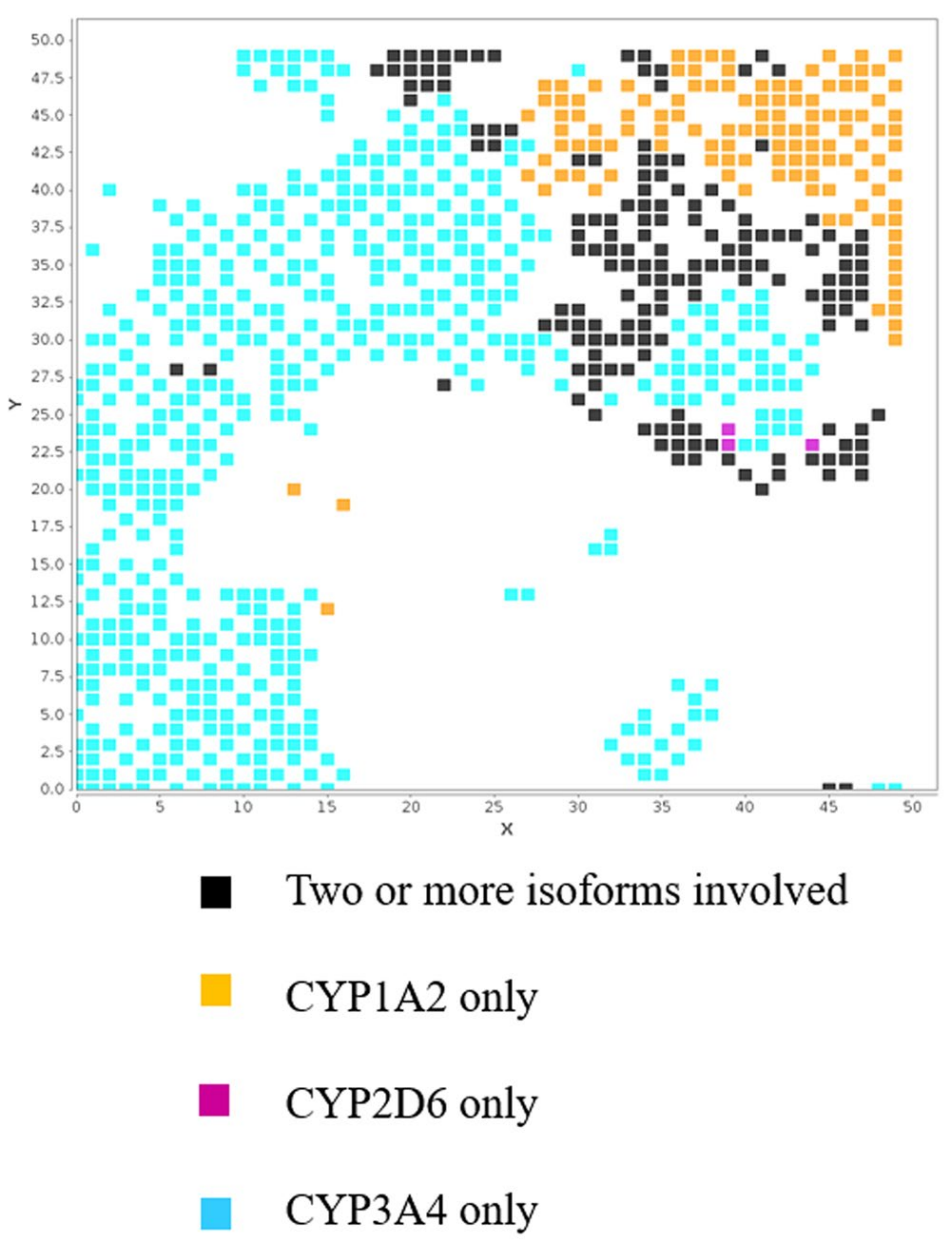
Combined map coloured by CYP implication. Only unstable nodes in the METASTAB human distribution are reported.

Drug metabolism is regulated by several enzymes, with cytochrome P450 often playing a key role. However, to know which CYP isoform is involved is as much important as it is to know whether CYP is involved or not. Figure 5 condenses the map of human metabolic stability with those of different CYP isoforms (1A2, 2D6 and 3A4, contribution to the metabolism). As in the previous example, only some regions are highlighted: those where molecules are predicted as unstable and with at least one CYP isoform “highly implicated”. Unsurprisingly, CYP3A4 (cyan nodes) is spread over a large part of the map, whereas CYP1A2 (orange nodes) is responsible for the metabolism of compounds that lie in the top-right region of the map. Black nodes correspond to the case of two or more isoforms involved, whereas very few pink nodes are specific for CYP2D6.

Hence, this comparison informs on the overall rate of metabolism (high rate in this case) and on the number of enzymes involved (which colour). In the case of only one enzyme is involved in metabolism the risk of drug-drug interactions (by decreased exposure due to metabolism inhibition) is more relevant. On the other hand, the DDIs risk is lower when two or more enzymes are involved. Finally, when more than one enzyme is involved, SAR for in silico prediction of metabolites is much more hazarded. Of course, what Fig. 5 shows is a simplification of the problem, whereas the number of possible isoforms involved is higher and, even in this case, there are regions of mismatch. In particular, regions of the space exist where nodes are described by low metabolism but also high implication of one of the isoforms, 1A2, 2D6 or 3A4 (data not shown). This is not unexpected, given the kind of data used for the CYP implication models (percentage of clearance of a single isoform divided by the overall microsomal clearance). Considering time consuming experiments, our approach provides an alternative to speed up some solutions proposals.

### Application 3: focus on bioavailability and drug-drug interactions risk

**Figure 6:**
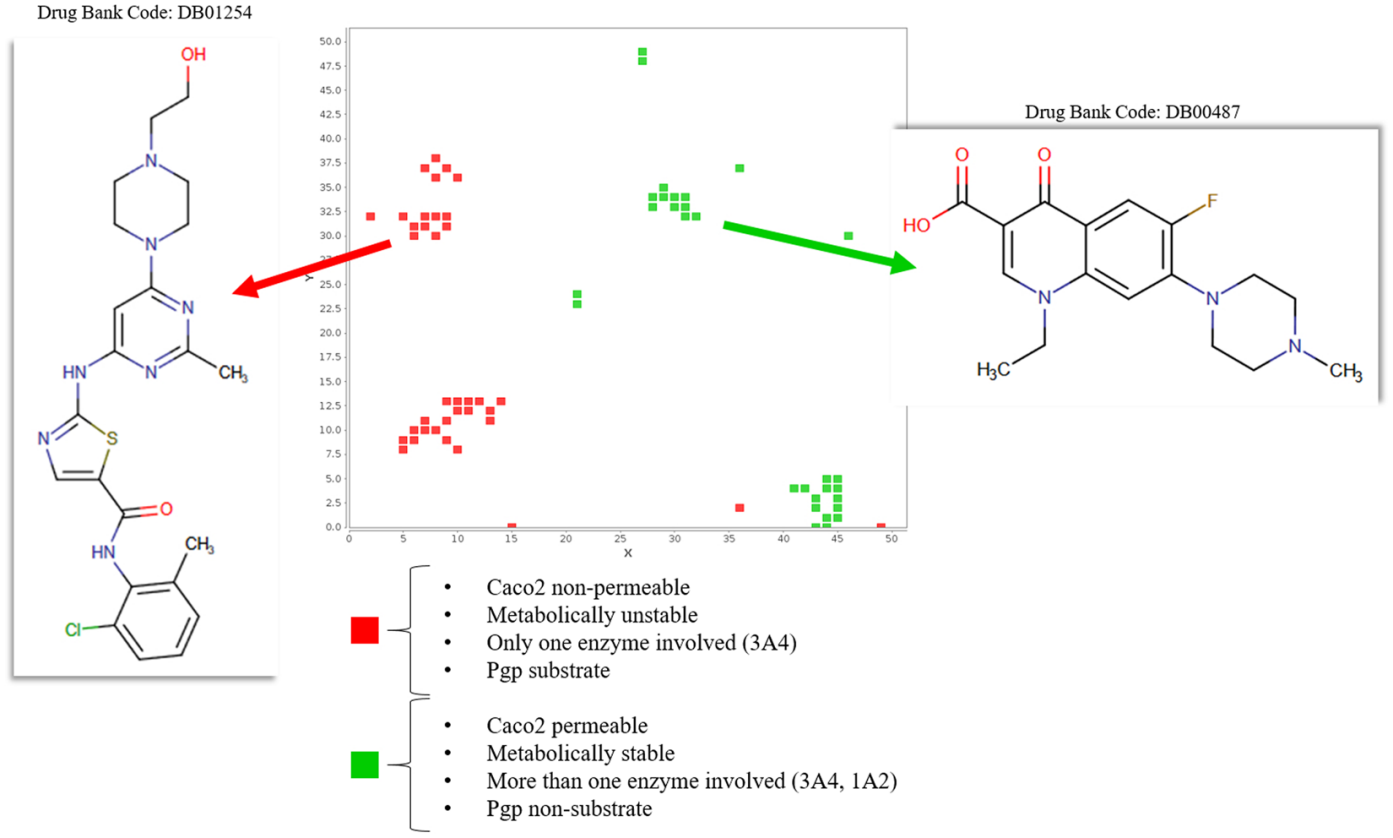
Regions of the ADME-Space with optimal (green) and non-optimal (red) ADME features.

The example reported in Fig. 6 concerns the detection in the space of regions with optimal (green) and non-optimal (red) ADME and DDI risk. To simplify, we considered only four features: Caco-2 permeability, human metabolic stability, Pgp recognition and the number of enzymes involved in metabolism, but the procedure can be repeated with even more properties. After combining the maps, we observe “risky” nodes (low absorption, low metabolic stability, implication of only one cytochrome and Pgp recognition), as well as “safe” nodes (high absorption, high metabolic stability, implication of more than one cytochrome and no Pgp recognition). The first two properties suggest the bioavailability of drugs, whilst the others can anticipate the risk of drug-drug interactions. Part of drug-drug interaction is due to the inhibition of drug metabolism by a co-administrated compound. Usually, the risk decreases when the number of enzymes involved in the metabolism of the drug increases. The probability of a complete blockage of the drug metabolism decreases when at least two or more metabolism enzymes are involved. If one of them is inhibited, the drug have a chance to be metabolized by the other. Whereas, when just one enzyme is involved, and it is inhibited, the drug-drug interaction risk may be important. Similarly, Pgp inhibition by a co-administrated compound increase the organism exposure to its substrates and, consequently, the risk of DDIs.

Concerning the just described case, in Fig. 6, two DrugBank molecules having these opposite features are reported. The information reported on DrugBank for DB01254 is that it is extensively metabolized, mainly by CYP3A4 and it is a Pgp substrate. For DB00487 the only information provided is the high intestinal absorption. Such information are in accordance with the ADME-Space maps.

Hence, a potential use of this kind of maps would be in screening, to guide the choice of molecular candidates: those predicted in red regions would be less preferred to those predicted in green regions, for either the expected low bioavailability or potentially higher drug-drug interaction risk.

### Application 4: Projecting Chemical Series

In order to study how the ADME-Space arranges structural analogues (molecules with minimal structural differences), 13 in dole-based molecules, recently objects of a SAR study^48^, were projected. After projection, 5 molecules (out of 13) were predicted in OFF cells, and were no further considered.

**Figure 7:**
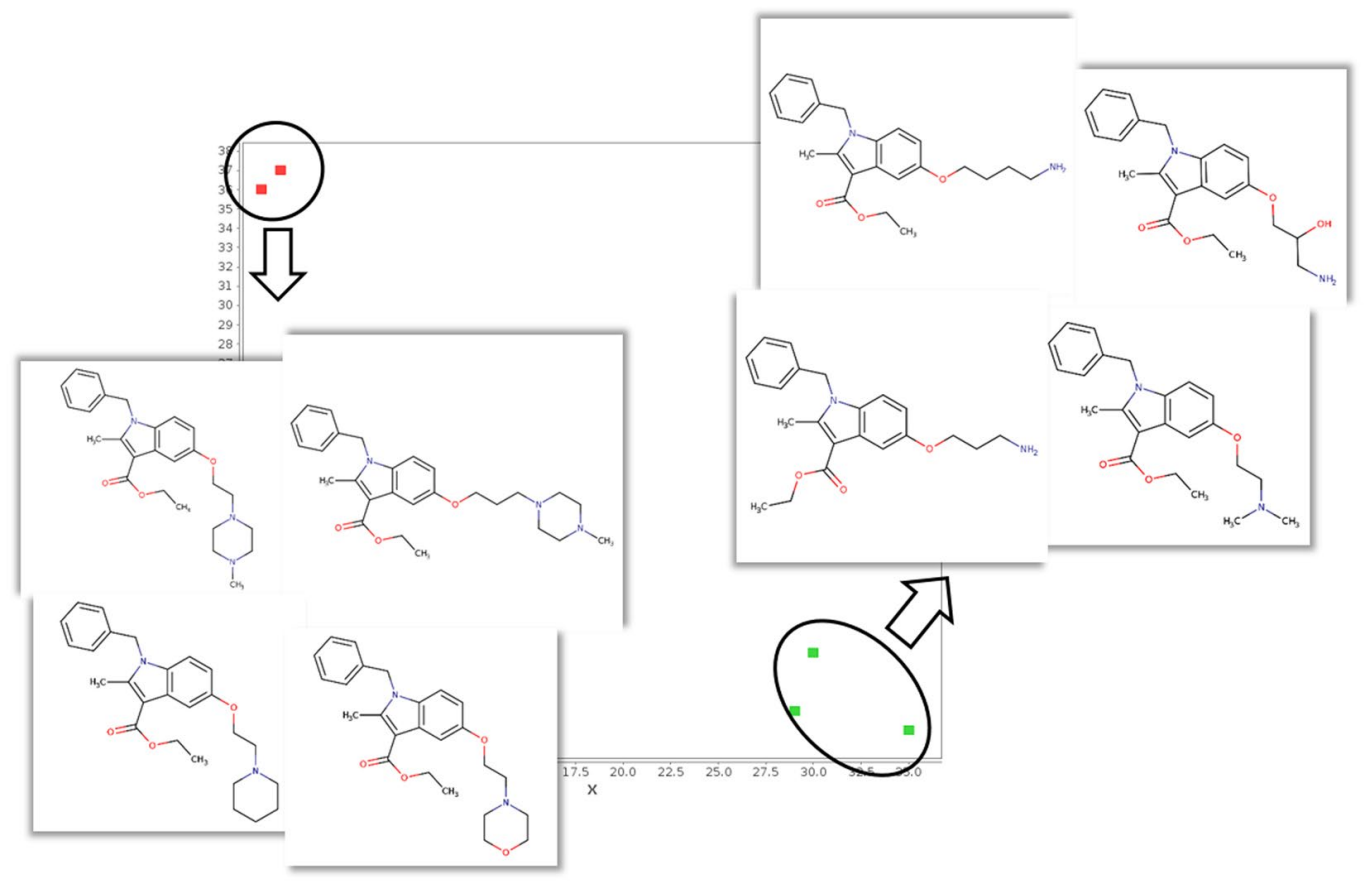
Spatial disposition of structurally similar molecules over the ADME-Space. Nodes are coloured by human metabolic stability (green: stable; red: unstable).

The disposition of the others is reported in Fig. 7. The top-left part of the ADME-Space allocates the four indoles with a ring moiety at the terminus of the side chain, whereas the derivatives without the ring moiety are located in the right-bottom part of the map. Furthermore, Fig. 7 shows how very similar molecules (based on the chemical structures), but with very different human metabolic stability will be grouped by their metabolic stability and not by their structure. This is the reason why two groups of molecules are emerging in very different regions of the space, one for low metabolic stability (red nodes) one for high metabolic stability (green nodes).

This means that In general, the model seems to consider similar, in terms of ADME, molecules with minor modifications (just one or two atoms are different). On the other hand, more relevant structural differences (like the addition of an aliphatic ring at the chain) cause important ADME-cliffs. As a result, molecules with approximately 80% of the structure in common, that in a fingerprint-based or VolSurf+-like based PCA would lie very closely, belong to nodes of the space that are far from each other. The fact that different chemical moieties may significantly alter the ADME profile of molecules is not new; what is noteworthy is that the space detected this alteration without any experimental check, confirming this method a useful tool in drug design.

### Application 5: Analysis of a new ADME property

**Figure 8:**
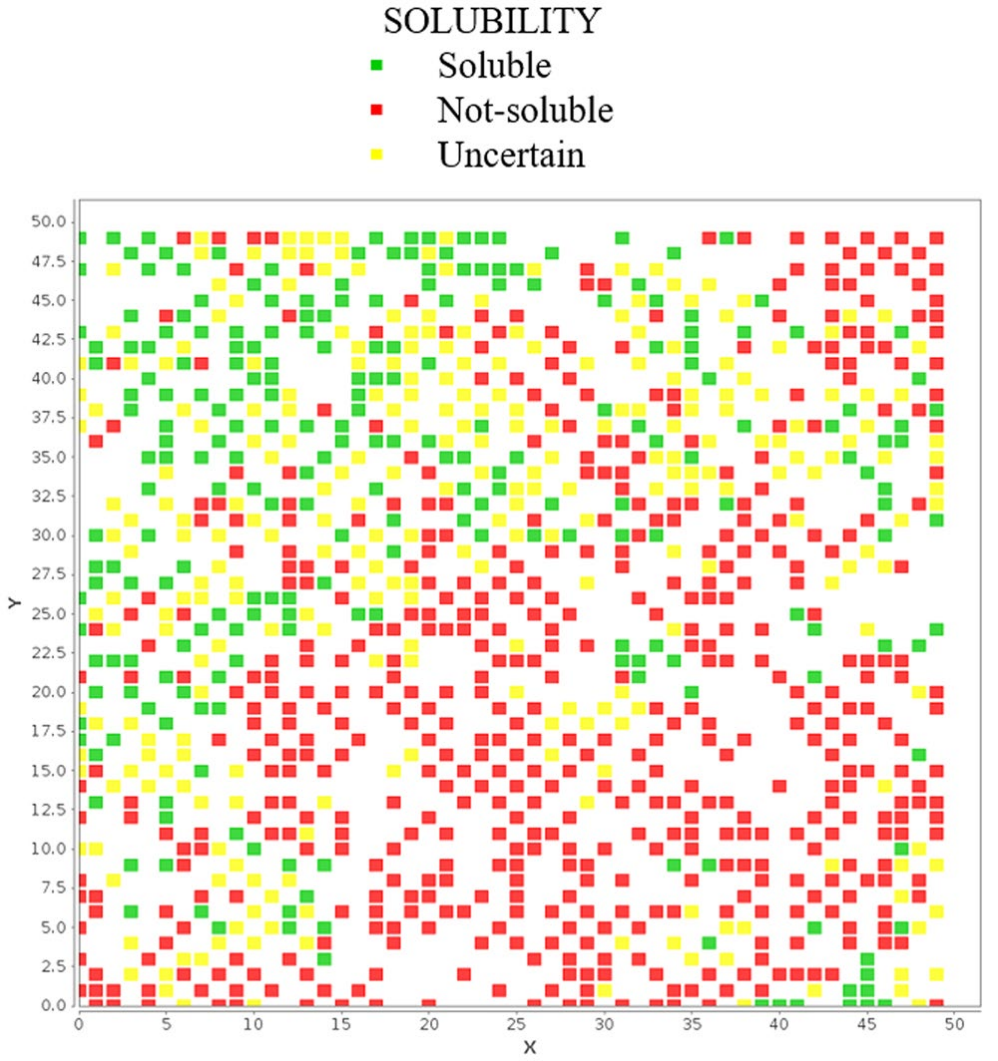
Region of the ADME-Space coloured by solubility.

We demonstrated here the ability of the ADME-Space to describe a naïve ADME property (which is not included in the ADME property used to create the map). We take the example of solubility experiments, measured on internal compounds by reprecipitation from DMSO stock solutions in buffer. The reported map shows clearly region of low solubility (red) and high solubility (green) (see Fig. 8).

This demonstrates the links between different ADME properties. Although solubility was not used, absorption, metabolism and transport properties are linked to solubility. This is the basis of the Biopharmaceutical Classification System (BCS) and Biopharmaceutics Drug Disposition Classification System (BDDCS) classification^49, 50^. When creating the map, compounds are grouped based on these ADME similarities. It is obvious that naive ADME properties linked to those used in the map building will appear as clear areas in the map. Our approach is a visual demonstration of these links through the ADME-Space. This ability of ADME-Space to anticipate naïve ADME properties, based on the ADME property similarity, is highly realistic and can be more effective if compared to classical pure chemical description used in classical chemical spaces. In our opinion, the use of ADME-Space as a support for compound ADME properties is a very powerful tool in visual data analysis of large datasets.

## Discussion and Conclusions

A big challenge for medicinal chemists is to design drugs with the desired biological properties (including expected activity and ADME profile), and to achieve it with the least number of attempts. In this perspective, predicting the ADME profile of drug candidates before their synthesis, in the early stage of drug discovery, could help in selecting candidates with the less critical ADME profile.

We propose here a computational tool approach, that we called ADME-Space: we wriggled form any structural description of the molecule, by focusing on its behaviour instead. By applying the SOM algorithm on approximately 26,000 molecules, each described by twenty predicted ADME properties, we obtained a two dimensional map where molecules are arranged according to their ADME behaviour. In other words, the novel procedure allows, for the first time to our knowledge, to project a molecule onto a space based on ADME properties. From the position on the map (the node), we get a trend on its ADME profile, because each node is associated to a specific ADME fingerprint. Moreover, the space developed is sensible to small structural modifications and it can be enriched with additional ADME properties.

In the ADME-Space, optimal regions can be defined for several ADME properties at the same time. In this way, the medicinal chemist can identify where optimum structures are supposed to be (this is not possible with chemical structure or fingerprint based spaces). Successively, it is possible to project the designed compounds and see where they are located compared to the expected optimal region (i.e. optimal compound). In addition, it is also possible to project libraries of compounds, see where there are located, and realize which structural modification could get closer to the optimal compound.

In conclusion, the ADME-Space opens a new framework for the multi-parametric data analysis in drug discovery. Projecting NMEs on this map is a new way to explain their behaviour, to explore the space, to look for the most suitable ADME profile, to get warnings on potential ADME problems, and even to choose the proper *in vitro* experiments to carry out. A perspective of this work can be the addition of the pharmacological dimension, which is an essential aspect in the research of new drugs. Moreover, the same identical procedure could be applied to public data (for example to DrugBank molecules) to develop an ADME-Space with public molecules and descriptors.

## Methods

### Experimental data curation: datasets from scientific literature

For P-glycoprotein inhibition, we used the dataset published by Broccatelli *et al*.^13^, who collected data for 1272 molecules from 61 articles. We used the data as originally conceived, with a training set of 772 molecules and a large test set of 503 molecules. The original model was based on a sequence of different “blocks”, composed by the molecular description obtained with the software VolSurf+ and Flap, as well as PLS and LDA as regression/classification methods. Here, we simplified the description (we used only VolSurf+ over the entire training set) but we treated the descriptors X-matrix by combining several classifiers.

For P-glycoprotein recognition, we used a dataset presented by Levatic *et al*.^27^, who classified compounds as P-glycoprotein substrates or nonsubstrates based on high-throughput data on different cancer cell lines. We used their dataset for modelling, composed of 934 molecules.

For the clearance model, we used the data collected by Lombardo F. and colleagues^10^, whose aim was to develop a computational model to predict the primary clearance mechanism, in order to guide further PK studies either *in vitro* or *in vivo*. They collected clearance data for 1028 molecules, but modelled a dataset of 469 with “clear quantitative data” for renal or metabolic clearance. They used a selection of VolSurf+ descriptors in combination with structural fragments, multivariate methods such as PCA and PLS, and an internal 5-fold cross-validation. We also limited the model to renal and metabolic pathways, but we used only VolSurf+ as descriptors and several supervised classifiers to build a composite model.

Contrera *et al*. from US Food and Drug Administration (FDA) compiled a database for the maximum recommended daily dose (MRDD) of 1309 pharmaceuticals and proposed some QSAR modelling based on MDL 2D-descriptors^26^. We downloaded the dataset from the FDA website, and developed a classification model by using only compounds with clear information on low/high MRDD (with low MRDD considered as potential toxic compounds and high MRDD as nontoxic compounds).

For the OCT2 inhibition model, we used the dataset proposed by Kido *et al*.^28^, who screened a library of 910 prescription drugs and drug-like compounds by using a high-throughput assay. Successively, they detected sub-clusters (i.e. substructures) of OCT2 inhibitors by means of the SOM algorithm applied to structural descriptors.

De Bruyn *et al*. presented an *in vitro* OATP1B inhibition high-throughput assay, to assess the inhibitory potential of drug candidates for the OATP1B protein^29^. They published data for 2000 molecules, for both 1B1 and 1B3 isoforms; after careful analysis (we observed a large overlap of the datasets), we decided to limit our modelling efforts to the isoform 1B1.

Concerning the BCRP datasets (inhibition and recognition), checking more than 100 publications led to the collection of 935 and 385 molecules, respectively. We kept only proved BCRP-binders, and we tuned the activity thresholds for compound categories comparing data for the same molecules, when available in two or more papers. Finally, both BCRP datasets were randomly split into training and test set (see details in Table 1). Details for BCRP-categories thresholds (based on IC_50_ and percentage of inhibition), as well as the collected data for BCRP recognition and BCRP inhibition is available as Supplementary Information Tables S8 and S9, whereas for the other datasets we refer to the original publications.

### Experimental data curation: private in-house datasets

In silico prediction of the Blood Brain Barrier (BRAIN PERM) permeability *in vivo* in rodent is based on experimental measurement of brain to plasma ratio of concentrations (Kp) *in vivo* at 2 time points after compound administration in rodents.

In silico prediction of Caco-2 Permeability (CACO2 PERM) was based on Caco-2 permeability assay which uses an established method for predicting the *in vivo* absorption of drugs across the gut wall by measuring the rate of transport of a compound across the Caco-2 cell line. The Caco-2 cell line is derived from a human colon carcinoma. The cells have characteristics that resemble intestinal epithelial cells such as the formation of a polarised monolayer, well-defined brush border on the apical surface and intercellular junctions. The absorbed fraction (Fabs) is the parameter that is modelled here.

In silico implication of human cytochrome P450 3A4 in the metabolism of drugs (CYP3A4 PERC) is based on experimental characterisation of the implication of Cyp 3A4 measured as the part of 3A4 in the Cyp P450 metabolism of drug (%Cyp3A4) compared to other Cyp metabolism in human (*in vitro* experiments based on incubation with bactosomes (transfection with human CYPs gene). The same is valid also for the isoforms 1A2 and 2D6.

In silico inhibition potential model is based on experimental measurements of IC_50_ of CYP2D6 enzyme. (CYP2D6 INHIB). A superzome is used to realise a competition between AMMC (3-[2-(N,N-Diethyl-N-methylammonium)ethyl]-7-methoxy-4methylcoumarin, a P450 activity probe) and a chemical (an inhibitor). The fluorescent metabolite of AMMC is measured by spectrofluometry at 3 concentrations of inhibitor: 25/2.5/0.25 µM. IC_50_ of the chemical is derived from the detection of fluorescence of the metabolite on blank, control and the 3 concentrations.

In silico inhibition potential model is based on experimental measurements of IC_50_ of CYP3A4 enzyme. A superzome is used to realise a competition between DBF (Dibenzylfluorescein, a P450 activity probe) and a chemical (an inhibitor). The fluorescent metabolite of DBF is measured by spectrofluometry at 3 concentrations of inhibitor: 25/2.5/0.25 µM. IC_50_ of the chemical is derived from the detection of fluorescence of the metabolite on blank, control and the 3 concentrations (CYP3A4 INHIB). The same procedure has been applied also for the isoforms 1A2 and 2C9.

In silico metabolic stability (METASTAB human, METASTAB rat, METASTAB mouse) is based on experimental metabolic bioavailability characterised *in vitro* in presence of corresponding species (human, rat, mouse) hepatic microsomes (10^−7^ M kinetic incubation up to 60 min with 0.33 mg prot/ml microsomal proteins).

Datasets were randomly divided in training and test sets and, wherever possible, the test set was split in an internal validation set (used for tuning the parameters) and an external (blind) validation set. The model for blood-brain ratio was the only exception: a robust PLS regression model was based on partition data between brain and plasma for a well-balanced training set (only 78 compounds).

### Data availability statement

Corporate datasets used and/or analysed during the current study are not publicly available due to non-patented research compounds. Public data used during this study are included in this published article (and its Supplementary Information files).

## Electronic supplementary material

Supplementary Information

Supplementary datasets
