# Supplementary material for: ADME-Space: a new tool for medicinal chemists to explore ADME properties
Source: Sci Rep. 2017 Jul 25;7:6359. doi: 10.1038/s41598-017-06692-0 (PMC5527008; doi:10.1038/s41598-017-06692-0)
Supplement: Supplementary file 1 — Supplementary Information [file 41598_2017_6692_MOESM1_ESM.pdf]

Supplementary Information

ADME-Space: a new tool for medicinal chemists to explore ADME properties

Giovanni Bocci,[1] Emanuele Carosati,*[1] Philippe Vayer,*[2] Alban Arrault,[2] Sylvain Lozano,[2] and Gabriele Cruciani[1]

[1]Laboratory of Chemometrics, Department of Chemistry, Biology and Biotechnology, University of Perugia, Via Elce di Sotto 8, 06123 Perugia, Italy

[2]Technologie Servier, 25-27 rue Eugène Vignat, BP 11749, 45007 Orléans cedex 1, France

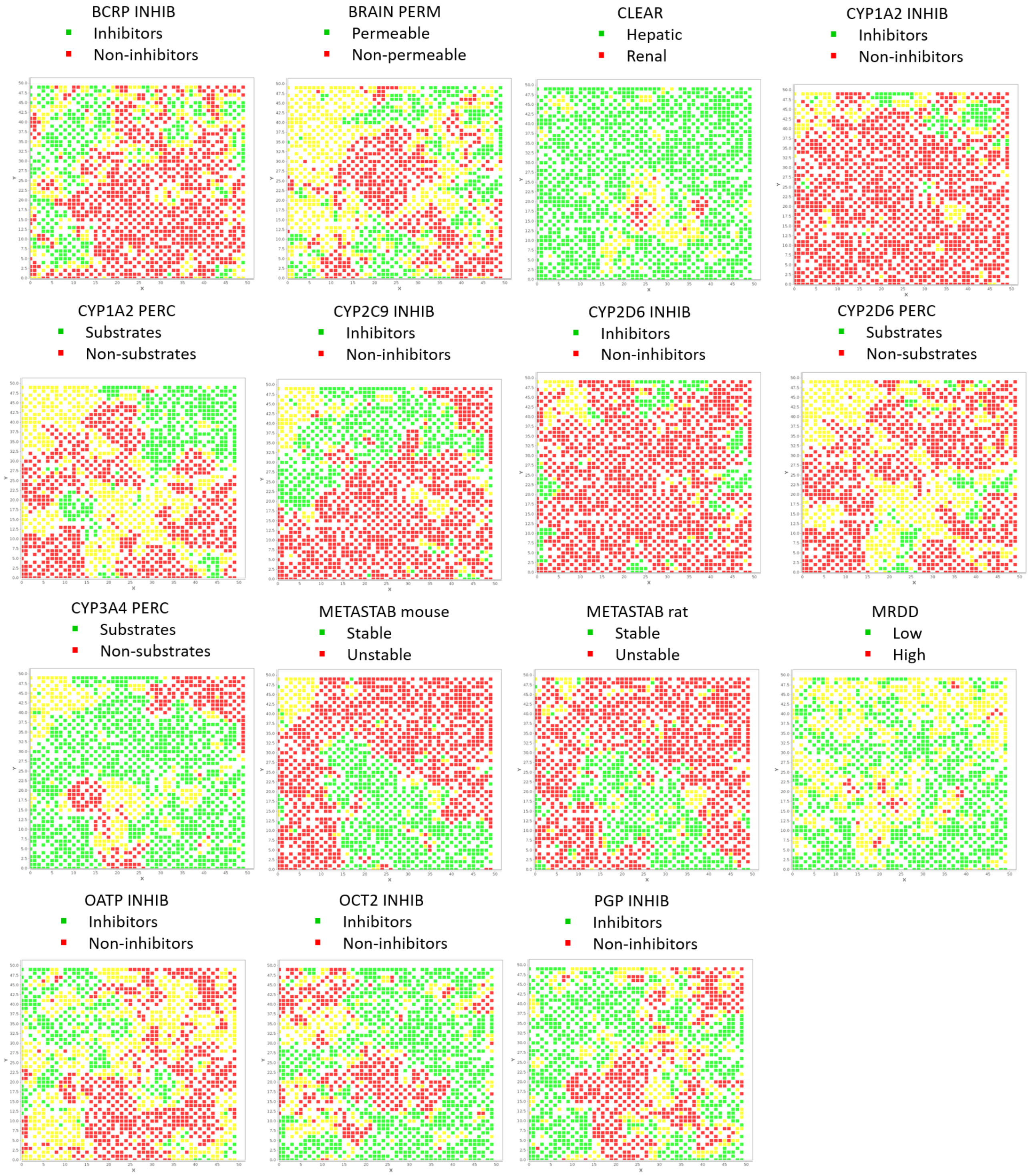

Figure S1: additional distribution plots for the ADME-Space properties. Uncertain nodes are coloured in yellow.

Table S1: Classifier parameters used for the PGP INHIB model

Classifier	Parameter	Value
RF	bootstrap	True
	class_weight	None
	criterion	gini
	max_depth	None
	max_features	auto
	max_leaf_nodes	None
	min_samples_leaf	1
	min_samples_split	2
	min_weight_fraction_leaf	0,0
	n_estimators	5
	n_jobs	1
	oob_score	True
	random_state	999
	verbose	0
	warm_start	False
SVM	C	1,0
	chach_size	200
	class_weight	None
	coef0	0,0
	degree	3
	gamma	0,03125
	kernel	rbf
	max_iter	-1,0
	probability	False
	random_state	None
	shrinking	True
	tol	0,001
	verbose	False
ETC	bootstrap	True
	class_weight	None
	criterion	gini
	max_depth	None
	max_features	auto
	max_leaf_nodes	None
	min_samples_leaf	1
	min_samples_split	2
	min_weight_fraction_leaf	0,0
	n_estimators	5
	n_jobs	1
	oob_score	False
	random_state	999
	verbose	0
	warm_start	False

AB	algorithm	SAMME.R
	base_estimator	None
	learning_rate	1,0
	n_estimators	20
	random_state	999
LDA	solver	svd
	priors	None
	n_components	None
	tol	0,01
	shrinkage	None
	store_covariance	False

Table S2: Classifier parameters used for the PGP RECOG model

Classifier	Parameter	Value
RF	bootstrap	True
	class_weight	None
	criterion	gini
	max_depth	None
	max_features	auto
	max_leaf_nodes	None
	min_samples_leaf	1
	min_samples_split	2
	min_weight_fraction_leaf	0,0
	n_estimators	5
	n_jobs	1
	oob_score	True
	random_state	999
	verbose	0
	warm_start	False
SVM	C	1,0
	chach_size	200
	class_weight	None
	coef0	0,0
	degree	3
	gamma	0,03125
	kernel	rbf
	max_iter	-1,0
	probability	False
	random_state	None
	shrinking	True
	tol	0,001
	verbose	False
ETC	bootstrap	True
	class_weight	None
	criterion	gini
	max_depth	None
	max_features	auto
	max_leaf_nodes	None
	min_samples_leaf	1
	min_samples_split	2
	min_weight_fraction_leaf	0,0
	n_estimators	5
	n_jobs	1
	oob_score	False
	random_state	999
	verbose	0
	warm_start	False

AB	algorithm	SAMME.R
	base_estimator	None
	learning_rate	1,0
	n_estimators	20
	random_state	999
LDA	solver	svd
	priors	None
	n_components	None
	tol	0,01
	shrinkage	None
	store_covariance	False
DT	class_weight	None
	criterion	gini
	max_depth	None
	max_features	None
	max_leaf_nodes	None
	min_sample_leaf	1
	min_sample_split	2
	min_weight_fraction_leaf	0
	random_state	None
	splitter	best

Table S3: Classifier parameters used for the BCRP INHIB model

Classifier	Parameter	Value
RF	bootstrap	True
	class_weight	None
	criterion	gini
	max_depth	None
	max_features	auto
	max_leaf_nodes	None
	min_samples_leaf	1
	min_samples_split	2
	min_weight_fraction_leaf	0,0
	n_estimators	25
	n_jobs	1
	oob_score	True
	random_state	999
	verbose	0
	warm_start	False
SVM	C	1,0
	chach_size	200
	class_weight	None
	coef0	0,0
	degree	3
	gamma	0,0
	kernel	rbf
	max_iter	-1,0
	probability	False
	random_state	None
	shrinking	True
	tol	0,001
	verbose	False
ETC	bootstrap	True
	class_weight	None
	criterion	gini
	max_depth	None
	max_features	auto
	max_leaf_nodes	None
	min_samples_leaf	1
	min_samples_split	2
	min_weight_fraction_leaf	0,0
	n_estimators	25
	n_jobs	1
	oob_score	False
	random_state	999
	verbose	0
	warm_start	False

AB	algorithm	SAMME.R
	base_estimator	None
	learning_rate	1,0
	n_estimators	100
	random_state	999
GB	init	None
	learning_state	0,1
	loss	deviance
	max_depth	3
	max_features	auto
	max_leaf_nodes	None
	min_samples_leaf	1
	min_samples_split	2
	min_weight_fraction_leaf	0,0
	n_estimators	100
	random_state	999
	subsample	1,0
	verbose	0
	warm_start	False

Table S4: Classifier parameters used for the BCRP RECOG model

Classifier	Parameter	Value
RF	bootstrap	True
	class_weight	None
	criterion	gini
	max_depth	7
	max_features	auto
	max_leaf_nodes	None
	min_samples_leaf	1
	min_samples_split	2
	min_weight_fraction_leaf	0,0
	n_estimators	30
	n_jobs	1
	oob_score	False
	random_state	999
	verbose	0
	warm_start	False
SVM	C	1,0
	chach_size	200
	class_weight	None
	coef0	0,0
	degree	3
	gamma	0,03125
	kernel	rbf
	max_iter	-1,0
	probability	False
	random_state	None
	shrinking	True
	tol	0,001
	verbose	False
ETC	bootstrap	False
	class_weight	None
	criterion	gini
	max_depth	None
	max_features	auto
	max_leaf_nodes	45
	min_samples_leaf	1
	min_samples_split	2
	min_weight_fraction_leaf	0,0
	n_estimators	35
	n_jobs	-1
	oob_score	False
	random_state	999
	verbose	0
	warm_start	False

Table S5: Classifier parameters used for the OCT2 INHIB model

Classifier	Parameter	Value
RF	bootstrap	True
	class_weight	None
	criterion	gini
	max_depth	None
	max_features	auto
	max_leaf_nodes	None
	min_samples_leaf	1
	min_samples_split	2
	min_weight_fraction_leaf	0,0
	n_estimators	25
	n_jobs	1
	oob_score	True
	random_state	999
	verbose	0
	warm_start	False
SVM	C	1,0
	chach_size	200
	class_weight	None
	coef0	0,0
	degree	3
	gamma	0,0
	kernel	rbf
	max_iter	-1,0
	probability	False
	random_state	None
	shrinking	True
	tol	0,001
	verbose	False
ETC	bootstrap	True
	class_weight	None
	criterion	gini
	max_depth	None
	max_features	auto
	max_leaf_nodes	None
	min_samples_leaf	1
	min_samples_split	2
	min_weight_fraction_leaf	0,0
	n_estimators	25
	n_jobs	1
	oob_score	False
	random_state	999
	verbose	0
	warm_start	False

AB	algorithm	SAMME.R
	base_estimator	None
	learning_rate	1,0
	n_estimators	100
	random_state	999
GB	init	None
	learning_state	0,1
	loss	deviance
	max_depth	3
	max_features	auto
	max_leaf_nodes	None
	min_samples_leaf	1
	min_samples_split	2
	min_weight_fraction_leaf	0,0
	n_estimators	100
	random_state	999
	subsample	1,0
	verbose	0
	warm_start	False

Table S6: Classifier parameters used for the CLEAR model

Classifier	Parameter	Value
RF	bootstrap	True
	class_weight	None
	criterion	gini
	max_depth	None
	max_features	auto
	max_leaf_nodes	None
	min_samples_leaf	1
	min_samples_split	2
	min_weight_fraction_leaf	0,0
	n_estimators	5
	n_jobs	1
	oob_score	True
	random_state	999
	verbose	0
	warm_start	False
SVM	C	1,0
	chach_size	200
	class_weight	None
	coef0	0,0
	degree	3
	gamma	0,03125
	kernel	rbf
	max_iter	-1,0
	probability	False
	random_state	None
	shrinking	True
	tol	0,001
	verbose	False
ETC	bootstrap	True
	class_weight	None
	criterion	gini
	max_depth	None
	max_features	auto
	max_leaf_nodes	None
	min_samples_leaf	1
	min_samples_split	2
	min_weight_fraction_leaf	0,0
	n_estimators	5
	n_jobs	1
	oob_score	False
	random_state	999
	verbose	0
	warm_start	False

AB	algorithm	SAMME.R
	base_estimator	None
	learning_rate	1,0
	n_estimators	20
	random_state	999
LDA	solver	svd
	priors	None
	n_components	None
	tol	0,01
	shrinkage	None
	store_covariance	False
DT	class_weight	None
	criterion	gini
	max_depth	None
	max_features	None
	max_leaf_nodes	None
	min_sample_leaf	1
	min_sample_split	2
	min_weight_fraction_leaf	0
	random_state	None
	splitter	best

Table S7: Classifier parameters used for the MRDD model

Classifier	Parameter	Value
RF	bootstrap	True
	class_weight	None
	criterion	gini
	max_depth	None
	max_features	auto
	max_leaf_nodes	None
	min_samples_leaf	1
	min_samples_split	2
	min_weight_fraction_leaf	0,0
	n_estimators	5
	n_jobs	1
	oob_score	True
	random_state	999
	verbose	0
	warm_start	False
SVM	C	1,0
	chach_size	200
	class_weight	None
	coef0	0,0
	degree	3
	gamma	0,03125
	kernel	rbf
	max_iter	-1,0
	probability	False
	random_state	None
	shrinking	True
	tol	0,001
	verbose	False
ETC	bootstrap	True
	class_weight	None
	criterion	gini
	max_depth	None
	max_features	auto
	max_leaf_nodes	None
	min_samples_leaf	1
	min_samples_split	2
	min_weight_fraction_leaf	0,0
	n_estimators	5
	n_jobs	1
	oob_score	False
	random_state	999
	verbose	0
	warm_start	False

LDA	solver	svd
	priors	None
	n_components	None
	tol	0,01
	shrinkage	None
	store_covariance	False
